# Metacognitive Strategies and Development of Critical Thinking in Higher Education

**DOI:** 10.3389/fpsyg.2022.913219

**Published:** 2022-06-15

**Authors:** Silvia F. Rivas, Carlos Saiz, Carlos Ossa

**Affiliations:** ^1^Departamento de Psicología Básica, Psicobiología y Metodología de CC, Facultad de Psicología, Universidad de Salamanca, Salamanca, Spain; ^2^Departamento de Ciencias de la Educación, Facultad de Educación y Humanidades, Universidad del Bío-Bío, Sede Chillán, Chile

**Keywords:** critical thinking, instruction, evaluation, metacognition, problem-solving

## Abstract

More and more often, we hear that higher education should foment critical thinking. The new skills focus for university teaching grants a central role to critical thinking in new study plans; however, using these skills well requires a certain degree of conscientiousness and its regulation. Metacognition therefore plays a crucial role in developing critical thinking and consists of a person being aware of their own thinking processes in order to improve them for better knowledge acquisition. Critical thinking depends on these metacognitive mechanisms functioning well, being conscious of the processes, actions, and emotions in play, and thereby having the chance to understand what has not been done well and correcting it. Even when there is evidence of the relation between metacognitive processes and critical thinking, there are still few initiatives which seek to clarify which process determines which other one, or whether there is interdependence between both. What we present in this study is therefore an intervention proposal to develop critical thinking and meta knowledge skills. In this context, Problem-Based Learning is a useful tool to develop these skills in higher education. The ARDESOS-DIAPROVE program seeks to foment critical thinking *via* metacognition and Problem-Based Learning methodology. It is known that learning quality improves when students apply metacognition; it is also known that effective problem-solving depends not only on critical thinking, but also on the skill of realization, and of cognitive and non-cognitive regulation. The study presented hereinafter therefore has the fundamental objective of showing whether instruction in critical thinking (ARDESOS-DIAPROVE) influences students’ metacognitive processes. One consequence of this is that critical thinking improves with the use of metacognition. The sample was comprised of first-year psychology students at Public University of the North of Spain who were undergoing the aforementioned program; PENCRISAL was used to evaluate critical thinking skills and the Metacognitive Activities Inventory (MAI) for evaluating metacognition. We expected an increase in critical thinking scores and metacognition following this intervention. As a conclusion, we indicate actions to incentivize metacognitive work among participants, both individually *via* reflective questions and decision diagrams, and at the interactional level with dialogues and reflective debates which strengthen critical thinking.

## Introduction

One of the principal objectives which education must cover is helping our students become autonomous and effective. Students’ ability to use strategies which help them direct their motivation toward action in the direction of the meta-proposal is a central aspect to keep at the front of our minds when considering education. This is where metacognition comes into play—knowledge about knowledge itself, a component which is in charge of directing, monitoring, regulating, organizing, and planning our skills in a helpful way, once these have come into operation. Metacognition helps form autonomous students, increasing consciousness about their own cognitive processes and their self-regulation so that they can regulate their own learning and transfer it to any area of their lives. As we see, it is a conscious activity of high-level thinking which allows us to look into and reflect upon how we learn and to control our own strategies and learning processes. We must therefore approach a problem which is increasing in our time, that of learning and knowledge from the perspective of active participation by students. To achieve these objectives of “learning to learn” we must use adequate cognitive learning strategies, among which we can highlight those oriented toward self-learning, developing metacognitive strategies, and critical thinking.

Metacognition is one of the research areas, which has contributed the most to the formation of the new conceptions of learning and teaching. In this sense, it has advanced within the constructivist conceptions of learning, which have attributed an increasing role to student consciousness and to the regulation which they exercise over their own learning (Glaser, 1994).

Metacognition was initially introduced by John Flavell in the early 1970s. He affirmed that metacognition, on one side, refers to “the knowledge which one has about his own cognitive processes products, or any other matter related with them” and on the other, “to the active supervision and consequent regulation and organization of these processes in relation with the objects or cognitive data upon which they act” (Flavell, 1976; p. 232). Based on this, we can differentiate two components of metacognition: one of a declarative nature, which is metacognitive knowledge, referring to knowledge of the person and the task, and another of a procedural nature, which is metacognitive control or self-regulated learning, which is always directed toward a goal and controlled by the learner.

Different authors have pointed out that metacognition presents these areas of thought or skills, aimed knowledge or toward the regulation of thought and action, mainly proposing a binary organization in which attentional processes are oriented, on occasions, toward an object or subject, and the other hand, toward to interact with objects and/or subjects (Drigas and Mitsea, 2021). However, it is possible to understand metacognition from another approach that establishes more levels of use of metacognitive thinking to promote knowledge, awareness, and intelligence, known as the eight pillars of metacognition model (Drigas and Mitsea, 2020). These pillars allow thought to promote the use of deep knowledge, cognitive processes, self-regulation, functional adaptation to society, pattern recognition and operations, and even meaningful memorization (Drigas and Mitsea, 2020).

In addition to the above, Drigas and Mitsea’s model establishes different levels where metacognition could be used, in a complex sequence from stimuli to transcendental ideas, in which each of the pillars could manifest a different facet of the process metacognitive, thus establishing a dialectical and integrative approach to learning and knowledge, allowing it to be understood as an evolutionary and complex process in stages (Drigas and Mitsea, 2021).

All this clarifies the importance of and need for metacognition, not only in education but also in our modern society, since this need to “teach how to learn” and the capacity to “learn how to learn” in order to achieve autonomous learning and transfer it to any area of our lives will let us face problems more successfully. This becomes a relevant challenge, especially today where it is required to have a broad view regarding reflection and consciousness, and to transcend simplistic and reductionist models that seek to center the problem of knowledge only around the neurobiological or the phenomenological scope (Sattin et al., 2021).

Critical thinking depends largely on these mechanisms functioning well and being conscious of the processes used, since this gives us the opportunity to understand what has not been done well and correct it in the future. Consciousness for critical thinking would imply a continuous process of reuse of thought, in escalations that allow thinking to be oriented both toward the objects of the world and toward the subjective interior, allowing to determine the ideas that give greater security to the person, and in that perspective, the metacognitive process, represents this use of Awareness, also allowing the generation of an identity of knowing being (Drigas and Mitsea, 2021).

We know that thinking critically involves reasoning and deciding to effectively solve a problem or reach goals. However, effective use of these skills requires a certain degree of consciousness and regulation of them. The ARDESOS-DIAPROVE program seeks precisely to foment critical thinking, in part, *via* metacognition (Saiz and Rivas, 2011, 2012, 2016).

However, it is not only centered on developing cognitive components, as this would be an important limitation. Since the 1990s, it has been known that non-cognitive components play a crucial role in developing critical thinking. However, there are few studies focusing on this relation. This intervention therefore considers both dimensions, where metacognitive processes play an essential role by providing evaluation and control mechanisms over the cognitive dimension.

### Metacognition and Critical Thinking

Critical Thinking is a concept without a firm consensus, as there have been and still are varying conceptions regarding it. Its nature is so complex that it is hard to synthesize all its aspects in a single definition. While there are numerous conceptions about critical thinking, it is necessary to be precise about which definition we will use. We understand that “*critical thinking* is a knowledge-seeking process *via* reasoning skills to solve problems and make decisions which allows us to more effectively achieve our desired results” (Saiz and Rivas, 2008, p. 131). Thinking effectively is desirable in all areas of individual and collective action. Currently, the background of the present field of critical thinking is also based in argumentation. Reasoning is used as the fundamental basis for all activities labeled as thinking. In a way, thinking cannot easily be decoupled from reasoning, at least if our understanding of it is “deriving something from another thing.” Inference or judgment is what we essentially find behind the concept of thinking. The question, though, is whether it can be affirmed that thinking is only reasoning. Some defend this concept (Johnson, 2008), while others believe the opposite, that solving problems and making decisions are activities which also form part of thinking processes (Halpern, 2003; Halpern and Dunn, 2021, 2022). To move forward in this sense, we will return to our previous definition. In that definition, we have specified intellectual activity with a goal intrinsic to all mental processes, namely, seeking knowledge. Achieving our ends depends not only on the intellectual dimension, as we may need our motor or perceptive activities, so it contributes little to affirm that critical thinking allows us to achieve our objectives as we can also achieve them by doing other activities. It is important for us to make an effort to identify the mental processes responsible for thinking and distinguish them from other things.

**Figure 1:**
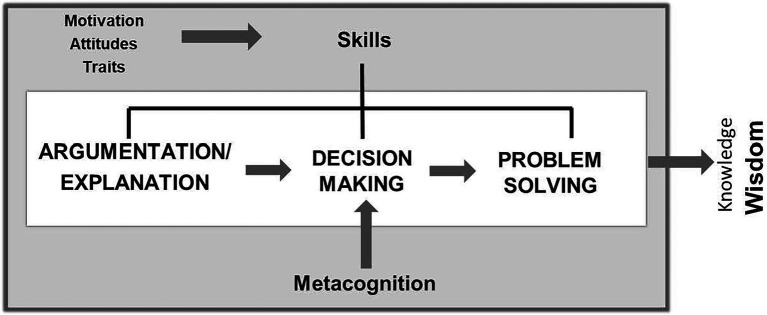
Components of critical thinking (Saiz, 2020).

Normally, we think to solve our problems. This is the second important activity of thought. A problem can be solved by reasoning, but also by planning course of action or selecting the best strategy for the situation. Apart from reasoning, we must therefore also make decisions to resolve difficulties. Choosing is one of the most frequent and important activities which we do. Because of this, we prefer to give it the leading role it deserves in a definition of thinking. Solving problems demands multiple intellectual activities, including reasoning, deciding, planning, etc. The final characteristic goes beyond the mechanisms peculiar to inference. What can be seen at the moment of delineating what it means to think effectively is that concepts are grouped together which go beyond the nuclear ideas of what has to do with inferring or reasoning. The majority of theoreticians in the field (APA, 1990; Ennis, 1996; Halpern, 1998, 2003; Paul and Elder, 2001; Facione, 2011; Halpern and Dunn, 2021, 2022) consider that, in order to carry out this type of thinking effectively, apart from having this skill set, the intervention of other types of components is necessary, such as metacognition and motivation. This is why we consider it necessary to speak about the components of critical thinking, as we can see in Figure 1:

In the nature of thinking, there are two types of components: the cognitive and the non-cognitive. The former include perception, learning, and memory processes. Learning is any knowledge acquisition mechanism, the most important of which is thinking. The latter refer to motivation and interests (attitudes tend to be understood as dispositions, inclinations…something close to motives); with metacognition remaining as a process which shares cognitive and non-cognitive aspects as it incorporates aspects of both judgment (evaluation) and disposition (control/efficiency) about thoughts (Azevedo, 2020; Shekhar and Rahnev, 2021). Both the cognitive and non-cognitive components are essential to improve critical thinking, as one component is incomplete without the other, that is, neither cognitive skills nor dispositions on their own suffice to train a person to think critically. In general, relations are bidirectional, although for didactic reasons only unidirectional relations appear in Figure 1 (Rivas et al., 2017). This is because learning is a dynamic process which is subject to all types of influence. For instance, if a student is motivated, they will work more and better—or at least, this is what is hoped for. If they can achieve good test scores as well, it can be supposed that motivation is reinforced, so that they will continue existing behaviors in the same direction that is, working hard and well on their studies. This latter point appears to arise at least because of an adjustment between expectations and reality which the student achieves thanks to metacognition, which allows them to effectively attribute their achievements to their efforts (Ugartetxea, 2001).

Metacognition, which is our interest in this paper, should also have bidirectional relations with critical thinking. Metacognition tends to be understood as the degree of consciousness which we have about our own mental processes and similar to the capacity for self-regulation, that is, planning and organization (Mayor et al., 1993). We observe that these two ideas have very different natures. The former is simpler, being the degree of consciousness which we reach about an internal mechanism or process. The latter is a less precise idea, since everything which has to do with self-regulation is hard to differentiate from a way of understanding motivation, such as the entire tradition of intrinsic motivation and self-determination from Deci, his collaborators, and other authors of this focus (see, e.g., Deci and Ryan, 1985; Ryan and Deci, 2000). The important thing is to emphasize the executive dimension of metacognition, more than the degree of consciousness, for practical reasons. It can be expected that this dimension has a greater influence on the learning process than that of consciousness, although there is little doubt that we have to establish both as necessary and sufficient conditions. However, the data must speak in this regard. Due to all of this, and as we shall see hereinafter, the intervention designed incorporates both components to improve critical thinking skills.

**Figure 2:**
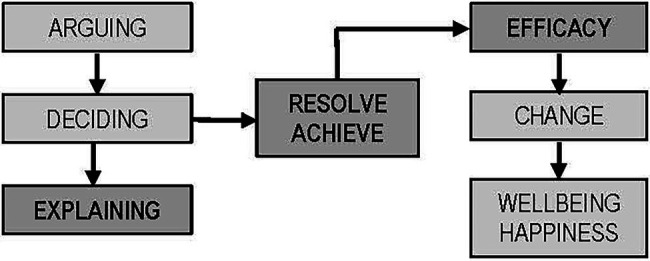
Purpose of critical thinking (Saiz, 2020, p.27).

We can observe, though, that the basic core of critical thinking continues to be topics related to skills, in our case, reasoning, problem-solving, and decision-making. The fact that we incorporate concepts of another nature, such as motivation, in a description of critical thinking is justified because it has been proven that, when speaking about critical thinking, the fact of centering solely on skills does not allow for fully gathering its complexity. The purpose of the schematic in Figure 2 is to provide conceptual clarity to the adjective “critical” in the expression *critical thinking*. If we understand *critical* to refer to *effective*, we should also consider that effectiveness is not, as previously mentioned, solely achieved with skills. They must be joined together with other mechanisms during different moments. Intellectual skills alone cannot achieve the effectiveness assumed within the term “critical.” First, for said skills to get underway, we must want to do so. Motivation therefore comes into play before skills and puts them into operation. For its part, metacognition allows us to take advantage of directing, organizing, and planning our skills and act once they have begun to work. Motivation thus activates our abilities, while metacognition lets them be more effective. The final objective should always be to gain proper knowledge of reality to resolve our problems.

We consider that the fact of referring to components of critical thinking while differentiating the skills of motivation and metacognition aids with the conceptual clarification we seek. On one side, we specify the skills which we discuss, and on another, we mention which other components are related to, and even overlap with them. We must be conscious of how difficult it is to find “pure” mental processes. Planning a course of action, an essential trait of metacognition, demands reflection, prediction, choice, comparison, and evaluation… And this, evidently, is thinking. The different levels or dimensions of our mental activity must be related and integrated. Our aim is to be able to identify what is substantial in thinking to know what we are able to improve and evaluate.

It is widely known that for our personal and professional functioning, thinking is necessary and useful. When we want to change a situation or gain something, all our mental mechanisms go into motion. We perceive the situation, identify relevant aspects of the problem, analyze all the available information, and appraise everything we analyze. We make judgments about the most relevant matters, decide about the options or pathways for resolution, execute the plan, obtain results, evaluate the results, estimate whether we have achieved our purpose and, according to the level of satisfaction following this estimation, consider our course of action good, or not.

The topic we must pose now is what things are teachable. It is useful to specify that what is acquired is clearly cognitive and some of the non-cognitive, because motivation can be stimulated or promoted, but not taught. The concepts of knowledge and wisdom are its basis. Mental representation and knowledge only become wisdom when we can apply it to reality, when we take it out of our mind and adequately situate it in the world. For our teaching purposes, we only have to take a position about whether knowledge is what makes critical thinking develop, or vice versa. For us, skills must be directly taught, and dominion is secondary. Up to now, we have established the components of critical thinking, but these elements still have to be interrelated properly. What we normally find are skills or components placed side by side or overlapping, but not the ways in which they influence each other. Lipman (2003) may have developed the most complete theory of critical and creative thinking, along Paul and his group, in second place, with their universal thought structures (Paul and Elder, 2006). However, a proposal for the relation between the elements is lacking.

To try to explain the relation between the components of thought, we will use Figure 2 as an aid.

The ultimate goal of critical thinking is change that is, passing from one state of wellbeing into a better state. This change is only the fruit of results, which must be the best. Effectiveness is simple achieving our goals in the best way possible. There are many possible results, but for our ends, there are always some which are better than others. Our position must be for effectiveness, the best response, the best solution. Reaching a goal is resolving or achieving something, and for this, we have mechanisms available which tell us which are the best course of action. Making decisions and solving problems are fundamental skills which are mutually interrelated. Decision strategies come before a solution. Choosing a course of action always comes before its execution, so it is easy to understand that decisions contribute to solutions.

Decisions must not come before reflection, although this often can and does happen. As we have already mentioned, the fundamental skills of critical thinking, in most cases, have been reduced to reasoning, and to a certain degree, this is justified. There is an entire important epistemological current behind this, within which the theory of argumentation makes no distinction, at least syntactically, between argumentation and explanation. However, for us this distinction is essential, especially in practice (Saiz, 2020). We will only center on an essential difference for our purpose. Argumentation may have to do with values and realities, but explanation only has to do with the latter. We can argue about beliefs, convictions, and facts, but we can only explain realities. Faced with an explanation of reality, any argumentation would be secondary. Thus, explanation will always be the central skill in critical thinking.

The change which is sought is always expressed in reality. Problems always are manifested and resolved with actions, and these are always a reality. An argument about realities aids in explaining them. An argument about values upholds a belief or a conviction. However, beliefs always influence behavior; thus, indirectly, the argument winds up being about realities. One may argue, for example, only for or against the death penalty, and reach the conviction that it is good or bad and ultimately take a position for or against allowing it. This is why we say that deciding always comes before resolving; furthermore, resolution always means deciding about something in a particular direction—it always means choosing and taking an option; furthermore, deciding is often only from two possibilities, the better or that which is not better, or which is not as good. Decisions are made based on the best option possible of all those which can be presented. Resolution is a dichotomy. Since our basic end lies within reality, explanation must be constituted as the basic pillar to produce change. Argumentation must therefore be at the service of causality (explanation), and both must be in the service of solid decisions leading us to the best solution or change of situation. We now believe that the relation established in Figure 2 can be better understood. From this relation, we propose that thinking critically means reaching the best explanation for an event, phenomenon, or problem in order to know how to effectively resolve it (Saiz, 2017, p.19). This idea, to our judgment, is the best summary of the nature of critical thinking. It clarifies details and makes explicit the components of critical thinking.

### Classroom Activities to Develop Metacognition

We will present a set of strategies to promote metacognitive work in the classroom in this section, aimed at improving critical thinking skills. These strategies can be applied both at the university level and the secondary school level; we will thus focus on these two levels, although metacognitive strategies can be worked on from an earlier age (Jaramillo and Osses, 2012; Tamayo-Alzate et al., 2019) and some authors have indicated that psychological maturity has a greater impact on effectively achieving metacognition (Sastre-Riba, 2012; García et al., 2016).

At the individual level, metacognition can be worked on *via* applying questions aimed at the relevant tasks which must be undertaken regarding a task (meta-knowledge questions), for example:

- Do I know how much I know about this subject?
- Do I have clear instructions and know what action is expected from me?
- How much time do I have?
- Am I covering the proper and necessary subjects, or is there anything important left out?
- How do I know that my work is right?
- Have I covered every point of the rubric for the work to gain a good grade or a sufficient level?

These reflective questions facilitate supervising knowledge level, resource use, and the final product achieved, so that the decisions taken for said activities are the best and excellent learning results are achieved.

Graphs or decision diagrams can also be used to aid in organizing these questions during the different phases of executing a task (planning, progress, and final evaluation), which is clearly linked with the knowledge and control processes of metacognition (Mateos, 2001). These diagrams are more complex and elaborate strategies than the questions, but are effective when monitoring the steps considered in the activity (Ossa et al., 2016). Decision diagrams begin from a question or task, detailing the principal steps to take, and associating an alternative (YES or NO) to each step, which leads to the next step whenever the decision is affirmative, or to improve or go further into the step taken if the decision is negative.

Finally, we can work on thinking aloud, a strategy which facilitates making the thoughts explicit and conscious, allowing us to monitor their knowledge, decisions, and actions to promote conscious planning, supervision and evaluation (Ávila et al., 2017; Dahik et al., 2019). For example:

- While asking a question, the student thinks aloud: *I am having problems with this part of the task, and I may have to ask the teacher to know whether I am right.*

Thinking aloud can be done individually or in pairs, allowing for active monitoring of decisions and questions arising from cognitive and procedural work done by the student.

Apart from the preceding strategies, it is also possible to fortify metacognitive development *via* personal interactions based on dialogue between both the students themselves and between the teacher and individual students. One initial strategy, similar to thinking out loud in pairs, is reflective dialogue between teacher and student, a technique which allows for exchanging deep questions and answers, where the student becomes conscious of their knowledge and practice thanks to dialogical interventions by the teacher (Urdaneta, 2014).

Reflective dialogue can also be done *via* reflective feedback implemented by the teacher for the students to learn by themselves about the positive and negative aspects of their performance on a task.

Finally, another activity based on dialogue and interaction is related to metacognitive argumentation (Sánchez-Castaño et al., 2015), a strategy which uses argumentative resources to establish a valid argumentative structure to facilitate responding to a question or applying it to a debate. While argumentative analysis is based on logic and the search for solid reasons, these can have higher or lower confidence and reliability as a function of the data which they provide. Thus, if a reflective argumentative process is performed, *via* questioning reasons or identifying counterarguments, there is more depth and density in the argumentative structure, achieving greater confidence and validity.

We can note that metacognition development strategies are based on reflective capacity, which allow thought to repeatedly review information and decisions to consider, without immediately taking sides or being carried away by superficial or biased ideas or data. Critical thought benefits strongly from applying this reflective process, which guides both data management and cognitive process use. These strategies can also be developed in various formats (written, graphic, oral, individual, and dialogical), providing teachers a wide range of tools to strengthen learning and thinking.

### Metacognitive Strategies to Improve Critical Thinking

In this section, we will describe the fundamental metacognitive strategies addressed in our critical thinking skills development program ARDESOS-DIAPROVE.

First, one of the active learning methodologies applied is Problem-Based Learning (PBL). This pedagogical strategy is student-centered and encourages autonomous and participative learning, orienting students toward more active and decisive learning. In PBL each situation must be approached as a problem-solving task, making it necessary to investigate, understand, interpret, reason, decide, and resolve. It is presented as a methodology which facilitates joint knowledge acquisition and skill learning. It is also good for working on daily problems *via* relevant situations, considerably reducing the distance between learning context and personal/professional life and aiding the connection between theory and practice, which promote the highly desired transference. It favors organization and the capacity to decide about problem-solving, which also improves performance and knowledge about the students’ own learning processes. Because of all this, this methodology aids in reflection and analysis processes, which in turn promotes metacognitive skill development.

The procedure which we carried out in the classroom with all the activities is based on the philosophy of gradual learning control transference (Mateos, 2001). During instruction, the teacher takes on the role of model and guide for students’ cognitive and metacognitive activity, gradually bringing them into participating in an increasing level of competency, and slowly withdrawing support in order to attain control over the students’ learning process. This methodology develops in four phases: (1) explicit instruction, where the teacher directly explains the skills which will be worked on; (2) guided practice, where the teacher acts as a collaborator to guide and aid students in self-regulation; and (3) cooperative practice, where cooperative group work facilitates interaction with a peer group collaborating to resolve the problem. By explaining, elaborating, and justifying their own points of view and alternative solutions, greater consciousness, reflection, and control over their own cognitive processes is promoted. Finally, (4) individual practice is what allows students to place their learning into practice in individual evaluation tasks.

Regarding the tasks, it is important to highlight that the activities must be aimed not only at acquiring declarative knowledge, but also at procedural knowledge. The objective of practical tasks, apart from developing fundamental knowledge, is to develop CT skills among students in both comprehension and expression in order to favor their learning and its transference. The problems used must be common situations, close to our students’ reality. The important thing in our task of teaching critical thinking is its usefulness to our students, which can only be achieved during application since we only know something when we are capable of applying it. We are not interested in students merely developing critical skills; they must also be able to generalize their intellectual skills, for which they must perceive them as useful in order to want to acquire them. Finally, they will have to actively participate to apply them to solving problems. Furthermore, if we study the different ways of reasoning without context, *via* overly academic problems, their application to the personal sphere becomes impossible, leading them to be considered hardly useful. This makes it important to contextualize skills within everyday problems or situations which help us get students to use them regularly and understand their usefulness.

Reflecting on how one carries things out in practice and analyzing mistakes are ways to encourage success and autonomy in learning. These self-regulation strategies are the properly metacognitive part of our study. The teacher has various resources to increase these strategies, particularly feedback oriented toward task resolution. Similarly, one of the most effective instruments to achieve it is using rubrics, a central tool for our methodology. These guides, used in student performance evaluations, describe the specific characteristics of a task at various performance levels, in order to clarify expectations for students’ work, evaluate their execution, and facilitate feedback. This type of technique also allows students to direct their own activity. We use them with this double goal in mind; on the one hand, they aid students in carrying out tasks, since they help divide the complex tasks they have to do into simpler jobs, and on the other, they help evaluate the task. Rubrics guide students in the skills and knowledge they need to acquire as well as facilitating self-evaluation, thereby favoring responsibility in their learning. Task rubrics are also the guide for evaluation which teachers carry out in classrooms, where they specify, review, and correctly resolve the tasks which students do according to the rubric criteria. Providing complete feedback to students is a crucial aspect for the learning process. Thus, in all sessions time is dedicated to carrying it out. This is what will allow them to move ahead in self-regulated skill learning.

According to what we have seen, there is a wide range of positions when it comes to defining critical thinking. However, there is consensus in the fact that critical thinking involves cognitive, attitudinal, and metacognitive components, which together favor proper performance in critical thinking (Ennis, 1987; Facione, 1990). This important relation between metacognition and critical thinking has been widely studied in the literature (Berardi-Coletta et al., 1995; Antonietti et al., 2000; Kuhn and Dean, 2004; Black, 2005; Coutinho et al., 2005; Orion and Kali, 2005; Schroyens, 2005; Akama, 2006; Choy and Cheah, 2009; Magno, 2010; Arslan, 2014) although not always in an applied way. Field studies indicate the existence of relations between teaching metacognitive strategies and progress in students’ higher-order thinking processes (Schraw, 1998; Kramarski et al., 2002; Van der Stel and Veenman, 2010). Metacognition is thus considered one of the most relevant predictors of achieving a complex higher-order thought process.

Along the same lines, different studies show the importance of developing metacognitive skills among students as it is related not only with developing critical thinking, but also with academic achievement and self-regulated learning (Klimenko and Alvares, 2009; Magno, 2010; Doganay and Demir, 2011; Özsoy, 2011). Klimenko and Alvares (2009) indicated that one way for students to acquire necessary tools to encourage autonomous learning is making cognitive and metacognitive strategies explicit and well-used and that teachers’ role is to be mediators and guides. Inspite of this evidence, there is less research about the use of metacognitive strategies in encouraging critical thinking. The principal reason is probably that it is methodologically difficult to gather direct data about active metacognitive processes which are complex by nature. Self-reporting is also still very common in metacognition evaluation, and there are few studies which have included objective measurements aiding in methodological precision for evaluating metacognition.

However, in recent years, greater importance has been assigned to teaching metacognitive skills in the educational system, as they aid students in developing higher-order thinking processes and improving their academic success (Flavell, 2004; Larkin, 2009). Because of this, classrooms have seen teaching and learning strategies emphasizing metacognitive knowledge and regulation. Returning to our objective, which is to improve critical thinking *via* the ARDESOS-DIAPROVE program, we have achieved our goal in an acceptable way (Saiz and Rivas, 2011, 2012, 2016).

However, we need to know which specific factors contribute to this improvement. We have covered significant ground through different studies, one of which we present here. In this one, we attempt to find out the role of metacognition in critical thinking. This is the central objective of the study. Our program includes motivational and metacognitive variables. Therefore, we seek to find out whether metacognition improves after this instruction program focused on metacognition. Therefore, our hypothesis is simple: we expect that the lesson will improve our students’ metacognition. The idea is to know whether applying metacognition helps us achieve improved critical thinking and whether after this change metaknowledge itself improves. In other words, improved critical thinking performance will make us think better about thinking processes themselves. If this can be improved, we can expect that in the future it will have a greater influence on critical thinking. The idea is to be able to demonstrate that applying specifically metacognitive techniques, the processes themselves will subsequently improve in quality and therefore contribute better volume and quality to reasoning tasks, decision-making and problem-solving.

## Materials and Methods

### Participants

In the present study, we used a sample of 89 students in a first-year psychology course at Public University of the North of Spain. 82% (73) were women, and the other 18% (16) were men. Participants’ median age was 18.93 (*SD* 1.744).

### Instruments

#### Critical Thinking Test

To measure critical thinking skills, we applied the *PENCRISAL* test (Saiz and Rivas, 2008; Rivas and Saiz, 2012). The *PENCRISAL* is a battery consisting of 35 production problem situations with an open-answer format, composed of five factors: *Deductive Reasoning*, *Inductive Reasoning*, *Practical Reasoning*, *Decision-Making*, and *Problem-Solving*, with seven items per factor. Items for each factor gather the most representative structures of fundamental critical thinking skills.

The items’ format is open, so that the person has to answer a concrete question, adding a justification for the reasons behind their answer. Because of this, there are standardized correction criteria assigning values between 0 and 2 points as a function of answer quality. This test offers us a total score of critical thinking skills and another five scores referring to the five factors. The value range is located between 0 and 72 points as a maximum limit for total test scoring, and between 0 and 14 for each of the five scales. The reliability measures present adequate precision levels according to the scoring procedures, with the lowest Cronbach’s alpha values at 0.632, and the test–retest correlation at 0.786 (Rivas and Saiz, 2012). PENCRISAL administration was done over the Internet *via* the evaluation platform SelectSurvey.NET V5: http://24.selectsurvey.net/pensamiento-critico/Login.aspx.

#### Metacognitive Skill Inventory

Metacognitive skill evaluation was done *via* the metacognitive awareness inventory from Schraw and Dennison (1994) (MAI; Huertas Bustos et al., 2014). This questionnaire has 52 Likert scale-type items with five points. The items are distributed in two general dimensions: cognitive knowledge (C) and regulation of cognition (R). This provides ample coverage for the two aforementioned ideas about metaknowledge. There are also eight defined subcategories within each general dimension. For C, these are: declarative knowledge (DK), procedural knowledge (PK), and conditional knowledge (CK). In R, we find: organization (O), monitoring (M), and evaluation (E). This instrument comprehensively, and fairly clearly, brings together essential aspects of metacognition. On one side, there is the level of consciousness, containing types of knowledge—declarative, procedural, and strategic. On the other, it considers everything important in the processes of self-regulation, planning, organization, direction or control (monitoring), adjustment (troubleshooting), and considering the results achieved (evaluation). It provides a very complete vision of everything important in this dimension. Cronbach’s alpha for this instrument is 0.94, showing good internal consistency.

### Intervention Program

As previously mentioned, in this study, we applied the third version of the ARDESOS_DIAPROVE program (Saiz and Rivas, 2016; Saiz, 2020), with the objective of improving thinking skills. This program is centered on directly teaching the skills which we consider essential to develop critical thinking and for proper performance in our daily affairs. For this, we must use reasoning and good problem-solving and decision-making strategies, with one of the most fundamental parts of our intervention being the use of everyday situations to develop these abilities.

DIAPROVE methodology incorporates three new and essential aspects: developing observation, the combined use of facts and deduction, and effective management of de-confirmation procedures, or discarding hypotheses. These are the foundation of our teaching, which requires specific teaching–learning techniques.

The intervention took place over 16 weeks and is designed to be applied in classrooms over a timeframe of 55–60 h. The program is applied in classes of around 30–35 students divided into groups of four for classwork in collaborative groups, and organized into six activity blocks: (1) nature of critical thinking, (2) problem-solving and effectiveness, (3) explanation and causality, (4) deduction and explanation, (5) argumentation and deduction, and (6) problem-solving and decision-making. These blocks are assembled maintaining homogeneity, facilitating a global integrated skill focus which helps form comprehension and use of the different structures in any situation as well as a greater degree of ability within the domain of each skill.

Our program made an integrated use of problem-based learning (PBL) and cooperative learning (CL) as didactic teaching and learning strategies in the critical thinking program. These methodologies jointly exert a positive influence on the students, allowing them to participate more actively in the learning process, achieve better results in contextualizing content and developing skills and abilities for problem-solving, and improve motivation.

To carry out our methodology in the classrooms, we have designed a teaching system aligned with these directives. Two types of tasks are done: (1) comprehension and (2) production. The materials we used to carry out these activities are the same for all the program blocks. One key element in our aim of teaching how to think critically must be its usefulness to our students, which is only achieved through application. This makes it important to contextualize reasoning types within common situations or problems, aiding students to use them regularly and understand their usefulness. Our intention with the materials we use is to face the problems of transference, usefulness, integrated skills, and how to produce these things. Accordingly, the *materials used* for the tasks are: (1) common situations and (2) professional/personal problems.

The tasks which the students perform take place over a week. They work in cooperative groups in class, and then review, correct, and clarify together, promoting reflection on their achievements and errors, which fortifies metacognition. Students get the necessary feedback on the work performed which will help them progressively acquire fundamental procedural contents. Our goal here is that students become conscious of their own thought processes in order to improve them. In this way, *via* the dialogue achieved between teachers and students as well as between the students themselves in their cooperative work, metacognition is developed. For conscious performance of tasks, the students will receive *rubrics* for each and every task to guide them in their completion.

### Procedure

Application of the ARDESOS-DIAPROVE program was done across a semester in the Psychology Department of the Public University of the North of Spain. One week before teaching began; critical thinking and metacognition evaluations were done. This was also done 1 week after the intervention ended, in order to gather the second measurement for PENCRISAL and MAI. The timelapse between the pre-treatment and post-treatment measurements was 4 months. The intervention was done by instructors with training and good experience in the program.

### Design

To test our objective, we used a quasi-experimental pre-post design with repeated measurements.

### Statistical Analysis

For statistical analysis, we used the IBM SPSS Statistics 26 statistical packet. The statistical tools and techniques used were: frequency and percentage tables for qualitative variables, exploratory and descriptive analysis of quantitative variables with a goodness of fit test to the normal Gaussian model, habitual descriptive statistics (median, SD, etc.) for numerical variables, and Student’s *t*-tests for significance of difference.

## Results

To begin, a descriptive analysis of the study variables was carried out. Tables 1, 2 present the summary of descriptions for the scores obtained by students in the sample, as well as the asymmetry and kurtosis coefficients for their distribution.

**Table 1:** Description of critical thinking measurement (PENCRISAL).

Variables	*N*	Min.	Max.	Median	*SD*	Asym	Kurt.	K-S p-sig. (exact)
TOT_PRE	89	11	37	25.14	5.436	−0.257	−0.197	0.309
RD_PRE	89	0	8	2.97	1.815	0.279	−0.387	0.036
RI_PRE	89	2	14	4.21	1.627	2.77	13.98	0.000
RP_PRE	89	1	11	5.69	2.248	0.186	−0.370	0.302
TD_PRE	89	2	11	6.23	1.796	0.118	−0.169	0.067
SP_PRE	89	1	11	6.01	2.058	−0.447	−0.262	0.015
TOT_POST	89	16	42	32.62	5.763	−0.807	0.447	0.161
RD_POST	89	0	10	4.81	2.189	−0.069	−0.692	0.059
RI_POST	89	2	9	5.37	1.547	0.031	−0.287	0.016
RP_POST	89	0	12	8.27	2.295	−0.818	1.198	0.056
TD_POST	89	3	11	7.82	1.748	−0.540	0.117	0.033
SP_POST	89	2	10	6.68	1.812	−0.617	0.508	0.027

TOT_PRE, PENCRISAL pre-test; RD_PRE, Deductive reasoning pre-test; RI_PRE, Inductive reasoning pre-test; RP_PRE, Practical reasoning pre-test; TD_PRE, Decision making pre-test; SP_PRE, Problem solving pre-test; TOT_POST, PENCRISAL post-test; RD_ POST, Deductive reasoning post-test; RI_ POST, Inductive reasoning post-test; RP_ POST, Practical reasoning post-test; TD_ POST, Decision making post-test; SP_ POST, Problem solving post-test; Min, minimum, Max, maximum, Asym, asymmetry; and Kurt, kurtosis.

**Table 2:** Description of metacognition measurement (MAI).

Variables	*N*	Min.	Max.	Media	*SD*	Asym.	Kurt.	K-S p-sig (exact)
TOT_MAI_PRE	89	145	233	192.13	16.636	−0.071	0.275	0.557
Decla_PRE	89	22	37	30.58	3.391	−0.594	−0.152	0.055
Proce_PRE	89	9	19	14.52	2.018	−0.560	0.372	0.004
Condi_PRE	89	8	23	18.04	3.003	−0.775	0.853	0.013
CONO_PRE	89	44	77	63.15	6.343	−0.384	0.044	0.445
Plani_PRE	89	10	31	24.35	4.073	−0.827	0.988	0.008
Orga_PRE	89	26	48	38.20	4.085	−0.307	0.331	0.022
Moni_PRE	89	15	35	25.24	3.760	−0.436	0.190	0.005
Depu_PRE	89	14	25	20.71	2.144	−0.509	0.310	0.004
Eva_PRE	89	12	28	20.49	3.310	−0.178	−0.044	0.176
REGU_PRE	89	97	160	128.99	12.489	−0.070	0.043	0.780
OT_MAI_POST	89	138	250	197.65	17.276	−0.179	0.969	0.495
Decla_POST	89	23	39	31.21	3.492	−0.407	0.305	0.020
Proce_POST	89	8	20	15.24	2.116	−0.723	0.882	0.001
Condi_POST	89	0	24	18.85	2.874	−0.743	0.490	0.029
CONO_ POST	89	44	82	65.30	6.639	−0.610	1.014	0.153
Plani_ POST	89	12	33	25.51	3.659	−0.539	0.994	0.107
Orga_ POST	89	27	48	39.40	4.150	−0.411	0.053	0.325
Moni_ POST	89	17	35	26.44	3.296	−0.277	0.421	0.143
Depu_ POST	89	15	24	20.40	2.245	−0.214	−0.531	0.023
Eva_ POST	89	12	29	20.60	3.680	−0.083	−0.098	0.121
REGU_PRE	89	94	168	132.35	12.973	−0.227	0.165	0.397

TOT_MAI_PRE, MAI pre-test; Decla_PRE, Declarative pre-test; Proce_PRE, Procedural pre-test; Condi_PRE, Conditional pre-test; CONO_PRE, Knowledge pre-test; Plani_PRE, Planning pre-test; Orga_PRE, Organization pre-test; Moni_PRE, Monitoring pre-test; Depu_PRE, Troubleshooting pre-test; Eva_PRE, Evaluation pre-test; REGU_PRE, Regulation pre-test; TOT_MAI_POST, MAI post-test; Decla_ POST, Declarative post-test; Proce_ POST, Procedural post-test; Condi_ POST, Conditional post-test; CONO_ POST, Knowledge post-test; Plani_ POST, Planning post-test; Orga_POST, Organization post-test; Moni_ POST, Monitoring post-test; Depu_ POST, Troubleshooting post-test; Eva_ POST, Evaluation post-test; and REGU_ POST, Regulation post-test;

As we see in the description of all study variables, the evidence is that the majority of them adequately fit the normal model, although some present significant deviations which can be explained by sample size.

Next, to verify whether there were significant differences in the metacognition variable based on measurements before and after the intervention, we contrasted medians for samples related with Student’s *t*-test (see Table 3).

**Table 3:** Comparison of the METAKNOWLEDGE variable as a function of PRE-POST measurements.

Variables		*N*	*M*	*SD*	Mean Difference (CI 95%)	*T* value	gl.	p-sig. (bilateral)
TOT_MAI	Pre.	89	192.13	16.636	−8.152_−2.882	−4.161	88	0.000**
	Post.	89	197.65	17.276				
Decla	Pre.	89	30.58	3.391	−1.235_−0.023	−2.063	88	0.042*
	Post.	89	31.21	3.492				
Proce	Pre.	89	14.52	2.018	−1.210_−0.228	−2.911	88	0.005*
	Post.	89	15.24	2.116				
Condi.	Pre.	89	18.04	3.003	−1.416_−0.202	−2.65	88	0.010*
	Post.	89	18.85	2.874				
CONO	Pre.	89	63.15	6.343	−3.289_−1.025	−3.787	88	0.000**
	Post.	89	65.3	6.639				
Plan	Pre.	89	24.35	4.073	−1.742_−0.573	−3.934	88	0.000**
	Post.	89	25.51	3.659				
Orga	Pre.	89	38.2	4.085	−2.054_−0.350	−2.803	88	0.006*
	Post.	89	39.4	4.15				
Moni	Pre.	89	25.24	3.76	−1.924_−0.480	−3.308	88	0.001*
	Post.	89	26.44	3.296				
TS	Pre.	89	20.71	2.144	−0.159_−0.766	1.303	88	0.196
	Post.	89	20.4	2.245				
Eval	Pre.	89	20.49	3.31	−0.815_−0.613	−0.282	88	0.779
	Post.	89	20.6	3.68				
REGU	Pre.	89	128.99	12.489	−5.364_−1.356	−3.331	88	0.001*
	Post.	89	132.35	12.973				

* Significant at 5%.

** Significant at 1%.

The results show that there are significant differences in the metaknowledge scale total and in most of its dimensions, where all the post medians for both the scale overall and for the three dimensions of the knowledge factor (declarative, procedural, and conditional) are higher than the pre-medians. However, in the cognition regulation dimension, there are only significant differences in the total and in the planning, organization, and monitoring dimensions. The medians are also greater in the post-test than the pre-test. However, the troubleshooting and evaluation dimensions do not differ significantly after intervention.

Finally, for critical thinking skills, the results show significant differences in the scale total and in the five factors regarding the measurement time, where performance medians rise after intervention (see Table 4).

**Table 4:** Comparison of the CRITICAL THINKING variable as a function of PRE-POST measurements.

Variables		N	M	SD	Student’s *T*-test			
					Mean difference (CI 95%)	*T* value	gl.	p-sig. (bilateral)
TOT	Pre.	89	25.146	5.436	−8.720_−6.246	−12.023	88	0.000**
	Post.	89	32.629	5.763				
RD	Pre.	89	2.978	3.391	−2.298_−1.364	−7.794	88	0.000*
	Post.	89	4.809	3.492				
RI	Pre.	89	4.213	1.627	−1.608_−0.706	−5.097	88	0.000*
	Post.	89	5.371	1.547				
RP	Pre.	89	18.04	2.248	−1.416_−0.202	−10.027	88	0.000*
	Post.	89	18.85	2.295				
TD	Pre.	89	63.15	1.796	−3.083_−2.063	−6.54	88	0.000**
	Post.	89	65.3	1.748				
SP	Pre.	89	24.35	2.058	−1.135_−0.213	−2.906	88	0.005**
	Post.	89	25.51	1.812				

* Significant at 5%.

** Significant at 1%.

These results show how metacognition improves due to CT intervention, as well as how critical thinking also improves with metacognitive intervention and CT skills intervention. Thus, it improves how people think about thinking as well as about the results achieved, since metacognition supports decision-making and final evaluation about proper strategies to solve problems.

## Discussion and Conclusions

The general aim of our study was to know whether a critical thinking intervention program can also influence metacognitive processes. We know that our teaching methodology improves cross-sectional skills in argumentation, explanation, decision-making, and problem-solving, but we do not know if this intervention also directly or indirectly influences metacognition. In our study, we sought to shed light on this little-known point. If we bear in mind the centrality of how we think about thinking for our cognitive machinery to function properly and reach the best results possible in the problems we face, it is hard to understand the lack of attention given to this theme in other research. Our study aimed to remedy this deficiency somewhat.

As said in the introduction, metacognition has to do with consciousness, planning, and regulation of our activities. These mechanisms, as understood by many authors, have a blended cognitive and non-cognitive nature, which is a conceptual imprecision; what is known, though, is the enormous influence they exert on fundamental thinking processes. However, there is a large knowledge gap about the factors which make metacognition itself improve. This second research lacuna is what we have partly aimed to shrink here as well with this study. Our guide has been the idea of knowing how to improve metacognition from a teaching initiative and from the improvement of fundamental critical thinking skills.

Our study has shed light in both directions, albeit in a modest way, since its design does not allow us to unequivocally discern some of the results obtained. However, we believe that the data provide relevant information to know more about existing relations between skills and metacognition, something which has seen little contrast. These results allow us to better describe these relations, guiding the design of future studies which can better discern their roles. Our data have shown that this relation is bidirectional, so that metacognition improves thinking skills and vice versa. It remains to establish a sequence of independent factors to avoid this confusion, something which the present study has aided with to be able to design future research in this area.

As the results show, total differences in almost all metaknowledge dimensions are higher after intervention; specifically, we see how in the knowledge factor the declarative, procedural, and conditional dimensions improve in post-measurements. This improvement moves in the direction we predicted. However, the cognitive regulation dimension only shows differences in the total, and in the planning, organization, and regulation dimensions. We can see how the declarative knowledge dimensions are more sensitive than the procedural ones to change, and within the latter, the dimensions over which we have more control are also more sensitive. With troubleshooting and evaluation, no changes are seen after intervention. We may interpret this lack of effects as being due to how everything referring to evaluating results is highly determined by calibration capacity, which is influenced by personality factors not considered in our study. Regarding critical thinking, we found differences in all its dimensions, with higher scores following intervention. We can tentatively state that this improved performance can be influenced not only by interventions, but also by the metacognitive improvement observed, although our study was incapable of separating these two factors, and merely established their relation.

As we know, when people think about thinking they can always increase their critical thinking performance. Being conscious of the mechanisms used in problem-solving and decision-making always contributes to improving their execution. However, we need to go into other topics to identify the specific determinants of these effects. Does performance improve because skills are metacognitively benefited? If so, how? Is it only the levels of consciousness which aid in regulating and planning execution, or do other factors also have to participate? What level of thinking skills can be beneficial for metacognition? At what skill level does this metacognitive change happen? And finally, we know that teaching is always metacognitive to the extent that it helps us know how to proceed with sufficient clarity, but does performance level modify consciousness or regulation level of our action? Do bad results paralyze metacognitive activity while good ones stimulate it? Ultimately, all of these open questions are the future implications which our current study has suggested. We believe them to be exciting and necessary challenges, which must be faced sooner rather than later. Finally, we cannot forget the implications derived from specific metacognitive instruction, as presented at the start of this study. An intervention of this type should also help us partially answer the aforementioned questions, as we cannot obviate what can be modified or changed by direct metacognition instruction.

## Data Availability Statement

The original contributions presented in the study are included in the article/supplementary material; further inquiries can be directed to the corresponding author.

## Ethics Statement

Ethical review and approval was not required for the study on human participants in accordance with the local legislation and institutional requirements. The patients/participants provided their written informed consent to participate in this study.

## Author Contributions

SR and CS contributed to the conception and design of the study. SR organized the database, performed the statistical analysis, and wrote the first draft of the manuscript. SR, CS, and CO wrote sections of the manuscript. All authors contributed to the article and approved the submitted version.

## Funding

This study was partly financed by the Project FONDECYT no. 11220056 ANID-Chile.

## Conflict of Interest

The authors declare that the research was conducted in the absence of any commercial or financial relationships that could be construed as a potential conflict of interest.

## Publisher’s Note

All claims expressed in this article are solely those of the authors and do not necessarily represent those of their affiliated organizations, or those of the publisher, the editors and the reviewers. Any product that may be evaluated in this article, or claim that may be made by its manufacturer, is not guaranteed or endorsed by the publisher.
